# Integrated response of the zebrafish (*Danio rerio*) cardiovascular system to hypoxia acclimation

**DOI:** 10.1242/jeb.251606

**Published:** 2025-11-20

**Authors:** Elizabeth A. Manchester, Todd E. Gillis

**Affiliations:** Department of Integrative Biology, University of Guelph, Guelph, ON, Canada, N1G 2W1

**Keywords:** Hypoxia tolerance, Cardiac remodelling, Environmental eutrophication

## Abstract

Chronic hypoxia exposure of fish can cause remodelling of the gills as well as increases to haematocrit and haemoglobin binding affinity. There is less known, however, about how chronic hypoxia affects the structure and function of the heart. In the current study, zebrafish were exposed to moderate hypoxia for 7 weeks and then ultrasound was used to characterize cardiac function. We found that cardiac output of the hypoxia-acclimated fish was greater than that of the control fish during an acute hypoxia exposure. This difference was due, at least in part, to the higher cardiac stroke volume. Histological measurements demonstrated an increase in the cross-sectional area of the ventricle of hypoxia-exposed fish and this was supported by higher end diastolic area measurements made using ultrasound. These changes to the heart occurred in conjunction with an increase in haematocrit and the respiratory surface area of the gills, as well as an improved capacity of the fish to respond to a more severe acute hypoxia challenge. We also found an increase in the expression of the gene transcripts for *hif-1αa* and *vegfaa* at 24 h, 3 days and 8 days of hypoxia exposure, suggesting a rapid and consistent response. Our results suggest that, unlike normoxia-acclimated fish which demonstrate a decrease in cardiac output with acute hypoxia exposure, zebrafish acclimated to hypoxia maintain cardiac output when acutely exposed to hypoxia.

## Teaser

**Summary:** Hypoxia-acclimated zebrafish maintain cardiac output during acute hypoxia exposure as the result of an increase in stroke volume facilitated by an increase in the cross-sectional area of the ventricle.

## INTRODUCTION

Eutrophication caused by agricultural run-off into freshwater and marine environments is a global problem and a growing challenge for wild fish populations due to the formation of hypoxic zones in nearshore areas (Breitburg et al., 2018; Diaz and Rosenberg, 2008). These hypoxic zones have been demonstrated to be increasing in severity, duration and frequency (Sinha et al., 2017). In addition, changes in rainfall patterns caused by global climate change are predicted to further exacerbate this problem by increasing the movement of fertilizers from the land into aquatic ecosystems (Sinha et al., 2017). Predicting the impact of the growing threat of environmental hypoxia on fish populations requires an understanding of the biological response to low oxygen and of the capacity of individual species to acclimate to it. For example, an increase in the severity or duration of a eutrophication event in a lake year after year may select for hypoxia-tolerant species and increase the energy budgets of these species as a result of the costs of the associated remodelling response and then maintenance of the tissue. As remodelling can involve cardiac hypertrophy, which occurs through changes in gene expression, protein expression and either cellular hypertrophy or hyperplasia, such costs have the potential to be significant (Keen et al., 2016; Gillis et al., 2024). Understanding the species-specific cost and consequences of prolonged hypoxia exposure would be useful for the creation of predictive models to help manage the impacts of environmental eutrophication.

Environmental hypoxia is a challenge for water-breathing animals as it may decrease the level of oxygen being carried to the tissues, which in turn affects cellular energy budgets and ultimately impairs growth and reproduction (Jenny et al., 2016; Shi et al., 2022; Tellier et al., 2022). One physiological system in fish that demonstrates an almost immediate response to hypoxia is the cardiorespiratory system, with acute exposure to low oxygen causing hyperventilation of the gills as well as an increase in haematocrit (Abdallah et al., 2015; Cadiz et al., 2019). At the organ level, acute hypoxia exposure increases cardiac load, promotes bradycardia (a decrease in heart rate) (Joyce and Wang, 2022) and has been demonstrated to reduce overall cardiac output (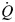) (Petersen and Gamperl, 2010). At the cellular level, 15 min of severe hypoxia (<20% air saturation) exposure has been reported to increase markers of cardiac oxidative stress, inflammation, cardiomyocyte apoptosis and tissue necrosis in zebrafish (Parente et al., 2013).

While acute exposure to hypoxia is a significant challenge for water-breathing fish, there is evidence that some species have the capacity to acclimate to hypoxic conditions with prolonged exposure. For example, Abdallah et al. (2015) have reported that aquatic surface respiration in zebrafish, initiated by an acute exposure, is reduced after 7 days of hypoxia, suggesting an increased capacity of the animals to obtain O_2_ from the environment and/or to move it to the tissues. Examples of physiological responses to chronic hypoxia exposure that could contribute to an acclimatory response include an increase in respiratory surface area of the gill to increase oxygen absorption (Dhillon et al., 2013; Mitrovic et al., 2009; Sollid et al., 2003), and an increase in blood oxygen affinity (Pan et al., 2017; Soivio et al., 1980). Work to characterize the response of the heart to hypoxia acclimation suggests that this is species dependent, with cardiac function being negatively affected with prolonged hypoxia exposure in intolerant species such as Atlantic cod (*Gadus morhua*) (Petersen and Gamperl, 2010), where 6–12 weeks of hypoxia (40% air saturation) caused a decrease in 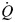 via a reduction in ventricular stroke volume (*v*_S_). In addition, Roberts et al. (2021) report that chronic hypoxia exposure of steelhead trout (*Oncorhynchus mykiss*) reduces myocardial shortening work and power, when measured in normoxia. In hypoxia-tolerant species, such as zebrafish and cichlids (*Haplochromis piceatus*), chronic hypoxia exposure (10% air saturation, 4 weeks) has been reported to cause an increase in myocardial density, atrophy of the outflow tract and significant changes to the heart transcriptome (Marques et al., 2008). In addition, the heart rate (*f*_H_) and opercular pressure of channel catfish (*Ictalurus punctatus*) acclimated to hypoxia for 7 days were higher than in control fish when measured during acute hypoxia exposure (Burleson et al., 2002). Together, these studies suggest that the functional capacity of the cardiovascular system is being actively modified during hypoxia acclimation. What is not known, however, is whether this remodelling response results in changes to cardiac output and whether this is related to manipulation of ventricle morphology.

The objective of the current study was to characterize the response of the cardiovascular system in a hypoxia-tolerant species to chronic hypoxia, specifically focusing on the structure and function of the heart. By integrating the use of histological approaches to investigate morphological changes and cardiac ultrasound to assess the functional impact of such morphological changes, we aimed to specifically examine the changes to heart morphology and functional capacity. This has not been previously done. We also wanted to determine whether acclimation to moderate hypoxia helps reduce the consequences of a subsequent, more severe acute hypoxia exposure. Such a response is relevant to determining whether fish in the natural environment can become tolerant to prolonged hypoxia exposure. To accomplish our experimental objectives, adult zebrafish were subjected to 7 weeks of moderate hypoxia (30% air saturation, 28°C) and then cardiovascular performance was assessed using a hypoxic loss of equilibrium trial where the fish were exposed to progressive, increasing levels of hypoxia. Cardiac ultrasound was also used to characterize *in vivo* heart function to determine whether hypoxia acclimation affected the functional response of the heart to acute hypoxia. Histological techniques were used to determine whether chronic hypoxia promotes structural remodelling of the gill and heart tissue. Finally, the expression of key gene transcripts used as markers for anaerobic metabolism, mitochondrial activity, angiogenesis and the hypoxia response were measured. Zebrafish were chosen for this study as, being endemic to the Ganges and Brahmaputra river basins in India, they inhabit slow-moving or standing water bodies where dissolved oxygen levels vary considerably as a result of seasonal changes in rainfall, plant growth and temperature (Das et al., 2022; Engeszer et al., 2007; Spence et al., 2008; Sundin et al., 2019). In addition, this species can survive moderate levels of hypoxia exposure in the lab (Cadiz et al., 2019; Mandic et al., 2020; Marques et al., 2008), and previous studies report that its cardiovascular system is phenotypically plastic in response to prolonged changes in environmental conditions (Johnson et al., 2014; Shaftoe et al., 2023). Thus, we hypothesized that acclimation of zebrafish to hypoxia promotes changes to the cardiovascular system that serve to increase transport capacity under hypoxic conditions. Further, we predicted that fish acclimated to chronic hypoxia will demonstrate improved cardiorespiratory performance in subsequent hypoxia exposure and exhibit distinct cardiac morphological characteristics.

## MATERIALS AND METHODS

### Experimental animals and housing

Approximately 300 male and female adult (11 months old) zebrafish, *Danio rerio* (F. Hamilton 1822), were acquired from the breeding programme at the University of Guelph. The mean (±s.e.m.) mass of these fish was 0.80±0.15 g and body length was 2.9±0.2 cm. These fish were then equally divided, keeping sex ratios even, into 12, 18.9 l aquaria so that there were 25 fish in each aquarium (treatment and control tank). There were 6 control tanks and 6 treatment tanks. Fish were randomly selected from these tanks for the different end points measured [ultrasound, loss of equilibrium (LOE) test, qPCR, histology]. These 12 aquaria were then split so that there were 6 tanks in each of the two ECARS (Environmentally Controlled Aquatic Recirculating Systems). One of these was the hypoxia treatment ECARS and the other the control ECARS. In each ECARS, separate water lines fed each aquarium from a central reservoir and the water drained from each aquarium via overflow, then passed through a common drain to the filter system for recirculation. All fish were held on a 12 h:12 h light:dark cycle and fed *ad libitum* with live brine shrimp each morning and Gemma 3000 powder each afternoon. Inflow water temperature was maintained at 28±1°C (Spence et al., 2006) via the control of the central recirculation system. Water parameters were maintained throughout the experiment, within recommended limits for zebrafish: ammonia <0.1 ppm, nitrite <0.02 ppm and nitrate <10 ppm (Aleström et al., 2020). All protocols were approved by the University of Guelph Animal Care Committee under the auspices of the Canadian Council for Animal Care.

### Exposure of zebrafish to chronic hypoxia acclimation

The aquaria in the control ECARS were maintained at ∼90% air saturation (138 mmHg) for the duration of the experiment. This is the oxygen saturation level of water when constantly aerated in the Hagen Aqualab. The dissolved oxygen (DO) of the aquaria in the hypoxia-acclimated ECARS was reduced to 30±1% air saturation (46 mmHg) and then maintained at that level for the duration of the experiment as described below (Abdallah et al., 2015; Marques et al., 2008; Shi et al., 2022). A DO level of 30% air saturation was chosen as previous research in zebrafish demonstrates that this is severe enough to promote hypoxia-induced physiological responses (Marques et al., 2008; Rees et al., 2001) but not below the partial pressure of oxygen (*P*_O_2__) at which O_2_ consumption can no longer be maintained in zebrafish (21 mmHg/32% oxygen saturation; Mandic et al., 2020). To create hypoxic water, nitrogen (N_2_) gas was bubbled into a header tank via a ceramic air stone. A second air stone bubbled compressed air into the same header tank. Oxygen saturation was maintained using a control system from Loligo Systems (Loligo^®^ Systems, Tjele, Denmark). A MINI-DO oxygen electrode constantly measured the DO of the header tank, and this sensor was attached to a solenoid valve that controlled the flow of N_2_ and air through their respective air stones. Additionally, sheets of foam insulation (Foamular NGX Rigid Foam Insulation) were floated on the exposed water surface of the ECARS and plastic bubble wrap was floated on the water surface of all aquaria to reduce O_2_ diffusion and prevent surface respiration.

### Timeline of experiment

Fish were allowed to acclimate to control conditions for 1 week, then the hypoxia system was turned on, depleting the DO levels to 30% air saturation at a rate of ∼2% air saturation per minute. A subset of animals was sampled and hearts were collected for RT-qPCR (*n*=10) at 24 h, 3 days and 8 days. All fish in this study were humanely euthanized via ice-water immersion then decapitation. The remaining zebrafish were held at their respective DO level for a total of 7 weeks. After this time, cardiorespiratory performance was measured via a hypoxic loss of equilibrium test (*n*=50 for both treatment and control), and cardiac function was characterized using high-frequency ultrasound (*n*=30 for both treatment and control). For histological measurements (*n*=10 fish for heart measurements and 6 fish for gill measurements), 5 sections were made per tissue (gill, heart) and 4–5 replicate measurements were made per section [gill lamellar length, interlamellar cell mass (ILCM), compact myocardium thickness, etc.]. The tissues of fish used in functional tests were not used for molecular analysis.

### High-frequency cardiac ultrasound imaging

Ultrasound imaging was performed following the 7 week experiment. Cardiac function was measured in control fish and those that had been chronically exposed to hypoxia under experimental conditions of normoxia at 28°C, hypoxia (20% air saturation) at 28°C, and normoxia at 20°C. All animals were fasted for 12 h prior to ultrasound imaging. Imaging was completed using a Vevo 3100 LT ultrasound (FUJIFILM Visual Sonics, Inc., Toronto, ON, Canada) as described by Shaftoe et al. (2023) with the following differences: fish were anaesthetized in system water at 28±1°C, containing a mixture of MS-222 (tricaine methanesulphonate, 0.15 g l^−1^, Syndel Canada) and isoflurane (0.5 ml l^−1^, Ontario Veterinary College, University of Guelph) and 0.2 g l^−1^ NaHCO_3_. This mixture was adapted from Collymore et al. (2014) to reduce the suppressant effects of MS222 on cardiovascular function. Once fish stopped responding to physical stimulation, they were transferred to the imaging stage. To characterize the influence of acute hypoxia exposure on cardiac function, the gills were perfused with hypoxic water, instead of normoxic water, that was drawn from a temperature-controlled reservoir containing an air stone bubbling N_2_. This water also contained the same concentrations of MS-222 and isoflurane as above. The oxygen levels of the reservoir were maintained at 20±2% air saturation and monitored using a FireSting Oxygen sensor (PyroScience sensor technology). The length and mass of the fish were recorded to standardize cardiac parameters (Wang et al., 2016) and to determine body condition using the equation for Fulton's body condition:

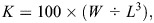
where *W* is the individual's body mass (g) and *L* represents the body length (cm).

### Analysis of ultrasound images

The ultrasound images were analysed using the Vevo 3100 LT image analysis software as previously described (Shaftoe et al., 2023). The parameters calculated were *v*_S_, ejection fraction, end-diastolic volume, end-systolic volume, ventricle area during systole and diastole (maximal relaxation), *f*_H_, peak velocity and ejection time. Heart rate was measured twice for each fish: first visually using the ultrasound during B-Mode imaging and then using Vevo analysis software through calculating the average of the intervals between aortic flow peaks. The average of these two measurements was then standardized to mass.

### LOE trials

The method used for the LOE trials was as described by Mandic et al. (2020). In brief, each group of fish (control or treatment, *n*=25) was transferred to the test tank 24 h prior to each trial. This tank was an 18.9 l aquarium containing an active air stone, inflow water and floating plastic plants for enrichment. The water in this tank was maintained at ∼90% air saturation and 28±1°C. Following this acclimation, the air stone was connected to a N_2_ cylinder via a standard gas regulator. DO in the aquarium was measured every second using a FireSting oxygen sensor. Bubble wrap was placed over the surface of the water to minimize surface respiration by the fish. Two trials were completed for each experimental group (control and acclimation) to generate *n*=50 trials for each group of 25 fish. Each trial was performed at 14:00 h every day for 4 days, and fish were fasted for the morning prior. At the start of each trial, the O_2_ flow to the tank was increased to allow the DO of the water to reach 100% air saturation and then held at this level for 10 min. This helped desensitize the fish to the active air stone. The DO was then rapidly decreased to 30% air saturation at a rate of −3.8% min^−1^, and then steadily (−0.13% min^−1^) to just below 4% air saturation when the test was completed. When an individual fish was no longer able to maintain position and floated to the surface, the time-to-loss-of-equilibrium (TLOE) and DO level (% air saturation) were recorded, the bubble wrap was pulled back, and the fish was transferred to an aerated recovery tank.

During the trials, video footage of zebrafish was captured to analyse gill ventilation rates. Videos were taken at four different levels of DO (50%, 30%, 20% and 15% air saturation) to estimate ventilation rates as oxygen was depleted. Work by Vulesevic et al. (2006) demonstrated that hypoxia exposure of zebrafish does not influence the amplitude of opercular displacement. This therefore makes measuring the rate of ventilation an appropriate method to measure changes in gill ventilation. The videos were slowed so that gill opercula movement could be counted for a 10 s period.

The effect of hypoxia exposure on TLOE was assessed by fitting a probability curve to model equilibrium probability against test time using a Kaplan–Meier survival test followed by a Mantel–Cox test (Claireaux et al., 2013). To interpret results quantitatively, differences in the median DO at LOE for each treatment were compared using a two-way analysis of variance (ANOVA) and Tukey's multiple comparisons test.

### Measurement of haematocrit

Haematocrit was measured as described by Eames et al. (2010). In brief, fish were euthanized by immersion in ice water and then the tail was cut off using scissors, just above the caudal keel. Blood was then collected from the wound using a pre-heparinized 75 mm microhaematocrit capillary tube (Fisher Scientific Co., Pittsburgh, PA, USA). The amount of blood collected per fish varied from 5 to 10 µl. The collection tubes were centrifuged at 13,700 ***g*** for 20 min. A ruler was used to measure the separated components of the blood capillary tube to calculate percentage haematocrit.

### Histological analysis of heart tissue

Zebrafish thoraxes were prepared for histological analysis as previously described (Johnson et al., 2014; Shaftoe et al., 2023) and then a microtome was used to make 7 µm sections. It should be noted that the hearts were washed in 1 mol l^−1^ KCl upon dissection to cause maximal contraction prior to fixation. This was done to ensure all hearts were in the same contractile state for morphological measurements. The sections were robotically stained for collagen and muscle using Picrosirius Red stain (Electron Microscopy Sciences, Hatfield, PA, USA) (Rich and Whittaker, 2005). Brightfield images of the ventricle and surrounding pericardial membrane were taken using a Nikon Ti Microscope. Five replicate sections were generated per individual. These images were used to determine ventricular area, collagen proportion and compact layer thickness. The average thickness of the compact layer was determined using ImageJ software at four random locations from five sections per heart as described by Johnson et al. (2014). The amount of collagen in the compact and spongy myocardium was determined from the same sections (Johnson et al., 2014; Rich and Whittaker, 2005). The micrograph images were transformed into their hue components using ImageJ and the colour threshold function yielded muscle (threshold=∼100) and collagen (threshold=13) densities (Rich and Whittaker, 2005).

### Histological analysis of gill tissue

After processing the zebrafish thoraxes, we removed the gill arches on the left side of each fish and embedded them in paraffin. A microtome was used to cut 5 µm sections, and the resulting slides were robotically stained with a standard haematoxylin and eosin (H&E) staining technique. Each individual was represented by 5 replicate sections. Micrographs were taken using a Nikon Ti Microscope. Lamellar length was quantified by measuring the distance from the base of the lamellae (protrusion from the gill filament) to the proximal tip. ILCM was measured by tracing the cross-sectional area of the cell mass. On each section, five measurements were taken for each trait (lamellar length and ILCM), and these were averaged to obtain a section-level average. The five section-level averages were then averaged to yield a final trait measurement per individual.

### qPCR

The zebrafish hearts were rinsed with physiological saline, dissected as described above and then snap frozen using dry ice. RNA was extracted, cDNA was generated and then qPCR was completed as previously described (Perugini et al., 2022). The genes quantified were: hypoxia inducible factor 1α (two paralogues: *hif-1ɑa* and *hif-1ɑb*), vascular endothelial growth factor Aa (*vegfaa*), cytochrome *c* oxidase (*cox4i1*) and lactate dehydrogenase (*ldh-b4*). The transcript levels of *ef1-ɑ* and *rpl8* were used as housekeeping genes, as the expression of these has been demonstrated to not be affected by hypoxia exposure (Alderman et al., 2019; Lim and Bernier, 2022). Please see Table S1 for the sequences of the primers used, efficiency values and *R*^2^ values. A non-RT control and a standard water control were included on each plate to ensure that the samples were not contaminated by genomic DNA or contamination in reagents, respectively.

### Statistical methods

All data were analysed using GraphPad Prism (ver. 10) and a significance threshold (α) of 0.05 was utilized. Differences between treatment groups and time points or experimental condition (i.e. data for qPCR, gill ventilation and heart functional parameters) were analysed using parametric two-way ANOVA, followed by a *post hoc* analysis (Tukey's HSD) if significant terms were found. Differences between treatment groups without interactive effects were analysed using Welch's *t*-test. Shapiro–Wilk tests were used to test the normality of the data, and Bartlett tests were used to verify homogeneity of variance. Outliers that were more than 2 times the standard deviation from the mean were removed from the dataset.

## RESULTS

### The effect of hypoxia acclimation on body condition and haematocrit

**Fig. 1.**
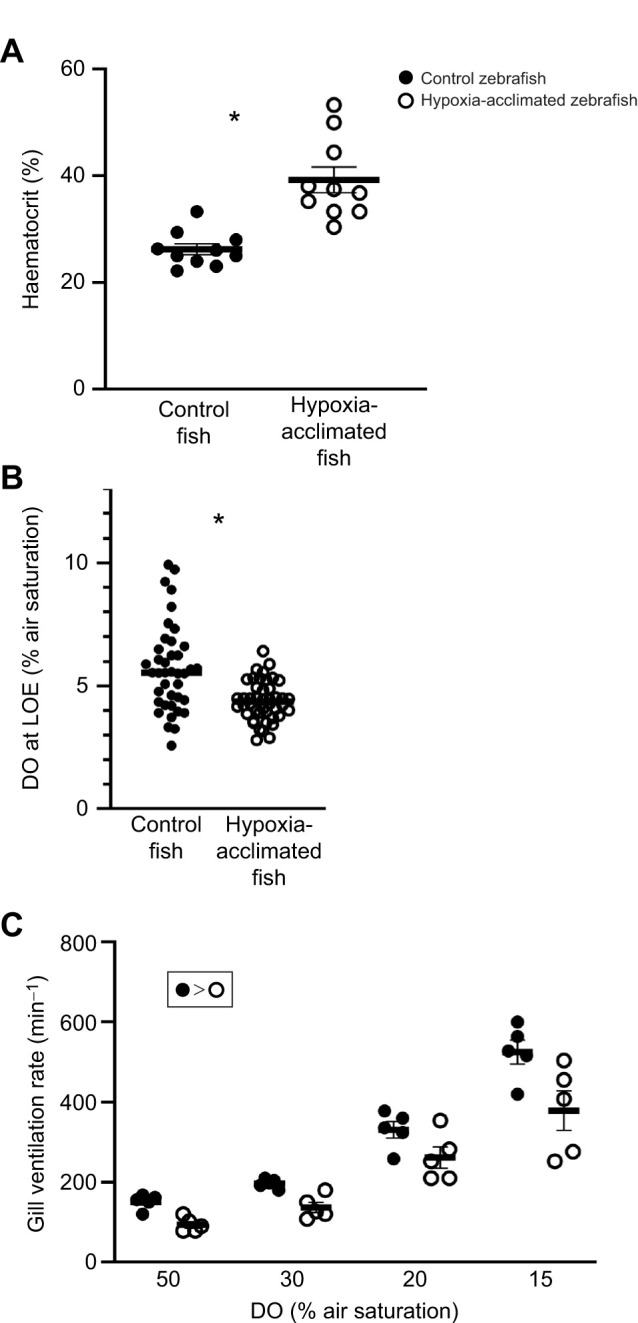
**Influence of hypoxia acclimation on cardiovascular parameters.** (A) Haematocrit, expressed as percentage packed red cell volume, of control fish and hypoxia-acclimated fish. (B) Dissolved oxygen (DO) levels, expressed as percentage air saturation, at which control fish and hypoxia-acclimated fish showed loss of equilibrium (LOE). (B) Gill ventilation rates of control fish and hypoxia-acclimated fish at 50%, 30%, 20% and 15% air saturation during an acute hypoxia exposure. Significant differences (*P*<0.05) are depicted using either an asterisk (between control and acclimated) or symbols (overall acclimation effect). Data are plotted as means±s.e.m., and individual data points are shown. For raw data, see Dataset 1.

There was no difference in the body condition of the control fish (1.71±0.04) from that of the hypoxia-acclimated fish (1.65±0.03) (*P*>0.05) (Fig. S1). The haematocrit of hypoxia-acclimated fish was ∼30% higher than that of control fish (*P*<0.0001, Fig. 1A).

### The effect of hypoxia acclimation on the physiological responses to acute hypoxia exposure

The hypoxia-acclimated zebrafish lost equilibrium at a significantly lower median DO than the control group (*P*<0.0001, Fig. 1B). This difference was equal to 1.31% air saturation and a time to LOE of 14 min. The results of the two-way ANOVA indicate that there was a significant effect of the level of DO (*P*<0.0001) and experimental treatment (hypoxia-acclimated or control group) on gill ventilation rate (*P*<0.0001). There was no significant interaction between the effects of acclimation and experimental conditions on gill ventilation rate (*P*=0.238). However, there was a significant effect of both DO (%) and experimental treatment on gill ventilation rate (*P*<0.0001). The average gill ventilation rate of the hypoxia-acclimated fish was lower than that of the control group (Fig. 1C).

### The effect of hypoxia acclimation on *in vivo* cardiac function

#### Heart rate

**Fig. 2.**
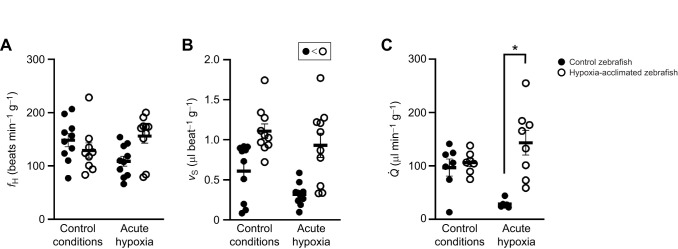
**Influence of hypoxia acclimation on *in vivo* cardiac function and how this is affected by acute hypoxia exposure.** (A) Heart rate (*f*_H_), (B) cardiac stroke volume (*v*_S_) and (C) cardiac output (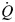). Significant differences (*P*<0.05; two-way ANOVA) are depicted using an asterisk (between control and acclimated), symbols (overall acclimation effect) or lowercase letters (within treatment group between experimental conditions). Data are plotted as means±s.e.m., and individual data points are shown. For raw data, see Dataset 1.

All cardiac functional measurements were normalized to body mass of the individual. There was a significant interaction between the effects of acclimation and experimental conditions on *f*_H_ (two-way ANOVA, *P*=0.004). Experimental treatment (hypoxia acclimation) had a significant impact on *f*_H_ (two-way ANOVA, *P*=0.0004), while experimental conditions (control, acute hypoxia or acute cold) did not (two-way ANOVA, *P*=0.9). *Post hoc* multiple comparisons analysis demonstrated that under control imaging conditions (normoxia, 28°C) and acute hypoxia (20% air saturation, 28°C), there was no significant difference in *f*_H_ between the hypoxia-acclimated group and the control group (*P*>0.05, Fig. 2A).

#### Stroke volume

There was no significant interaction between the effects of acclimation group and experimental conditions on *v*_S_ (two-way ANOVA, *P*=0.272). Experimental treatment (hypoxia acclimation) had a significant impact on *v*_S_ (two-way ANOVA, *P*<0.0001) (Fig. 2B). *Post hoc* analysis revealed that the *v*_S_ of hypoxia-acclimated fish was significantly higher than that of the control group. Under control conditions, acute hypoxia and acute cold conditions, the *v*_S_ of the treatment group was 1.8-, 2.9- and 1.6-fold higher than that of the control fish when measured under the same experimental conditions (two-way ANOVA, *P*=0.037).

#### Cardiac output

There was a significant interaction between the effects of acclimation group and experimental conditions on 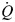 (two-way ANOVA, *P*=0.003). Experimental treatment (hypoxia acclimation) had the most significant impact on 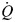 (two-way ANOVA, *P*<0.0001). Imaging conditions (control, acute hypoxia or acute cold) did not have a significant effect on 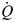 (two-way ANOVA, *P*=0.552). Under control imaging conditions (normoxia, 28°C), the 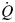 of hypoxia-acclimated fish did not differ significantly from that of control fish (*P*>0.05, Fig. 2C). The 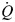 of control fish measured under acute hypoxia (20% air saturation, 28°C) was ∼60% lower than that measured under control conditions (*P*=0.032). There was, however, no difference in the 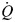 of the hypoxia-acclimated fish when measured under acute hypoxia or control conditions (*P*>0.05). Under acute hypoxia conditions, the 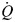 of hypoxia-acclimated fish was significantly higher than that of control fish (*P*<0.0001, Fig. 2C).

### The effect of hypoxia acclimation on heart size, morphology and composition

**Fig. 3.**
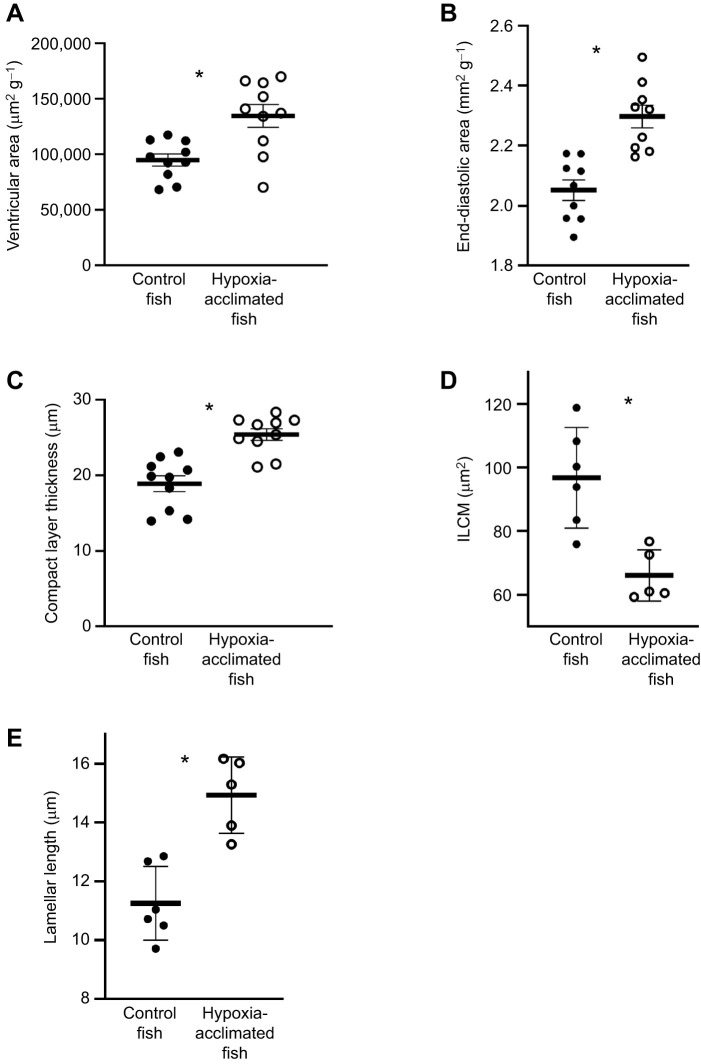
**Influence of hypoxia acclimation on cardiac ventricle and gill histology.** (A) Ventricular cross-sectional area, (B) end diastolic area, (C) compact layer thickness, (D) area of interlamellar cell mass (ILCM) and (E) lamellar length. Significant differences (*P*<0.05) are depicted using an asterisk (between control and acclimated). Data are plotted as means±s.e.m., and individual data points are shown. For raw data, see Dataset 1.

**Fig. 4.**
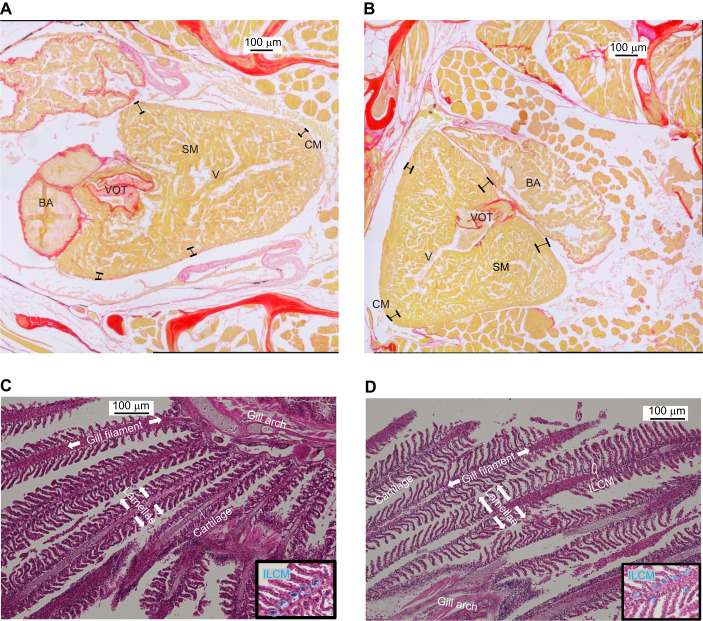
**Representative brightfield images of the heart and gill tissue from control and hypoxia-acclimated zebrafish.** (A) Cross-section of a control zebrafish heart at low magnification. (B) Cross-section of a hypoxia-acclimated zebrafish heart at high magnification. (C) Cross-section of a control zebrafish gill at high magnification. (D) Cross-section of a hypoxia-acclimated zebrafish gill at high magnification. Insets in C and D are magnified images showing highlighted ILCM. BA, bulbous arteriosus; SM, spongy myocardium; VOT, ventricular outflow tract; V, ventricle; CM, compact myocardium. Scale bars: 100 µm.

The average cross-sectional area of the cardiac ventricles from hypoxia-acclimated fish, standardized to body mass, was significantly greater than that of control fish (two-tailed *t*-test, *P*=0.003, Fig. 3A). The end-diastolic ventricular area of hypoxia-acclimated zebrafish, characterized by ultrasound under control conditions, was 1.1-fold that of the control fish (two-tailed *t*-test, *P*=0.0002, Fig. 3B). The thickness of the compact myocardium from the hearts of hypoxia-acclimated zebrafish was ∼69% greater than that of control fish (two-tailed *t*-test, *P*<0.0001, Figs 3C and 4A,B). Finally, the calculated area of collagen in the cross-sectional area of cardiac ventricles from hypoxia-acclimated fish standardized to the mass of the animal (1781.3±184.1 µm^−2^ g^−1^) was not different from that of control fish (1691.8±212.3 µm^−2^ g^−1^) (*P*>0.05) (Fig. S2).

### The effect of hypoxia acclimation on gill morphology

The average ILCM of gills from control fish was significantly (∼68%) greater than that of hypoxia-acclimated fish (two-tailed *t*-test, *P*=0.0035, Figs 3D and 4C,D). The average lamellar length of gills from hypoxia-acclimated fish was significantly greater than that of control fish (two-tailed *t*-test, *P*=0.001, Figs 3E and 4C,D).

### The effect of hypoxia acclimation on the expression of select genes

#### hif-1αa

**Fig. 5.**
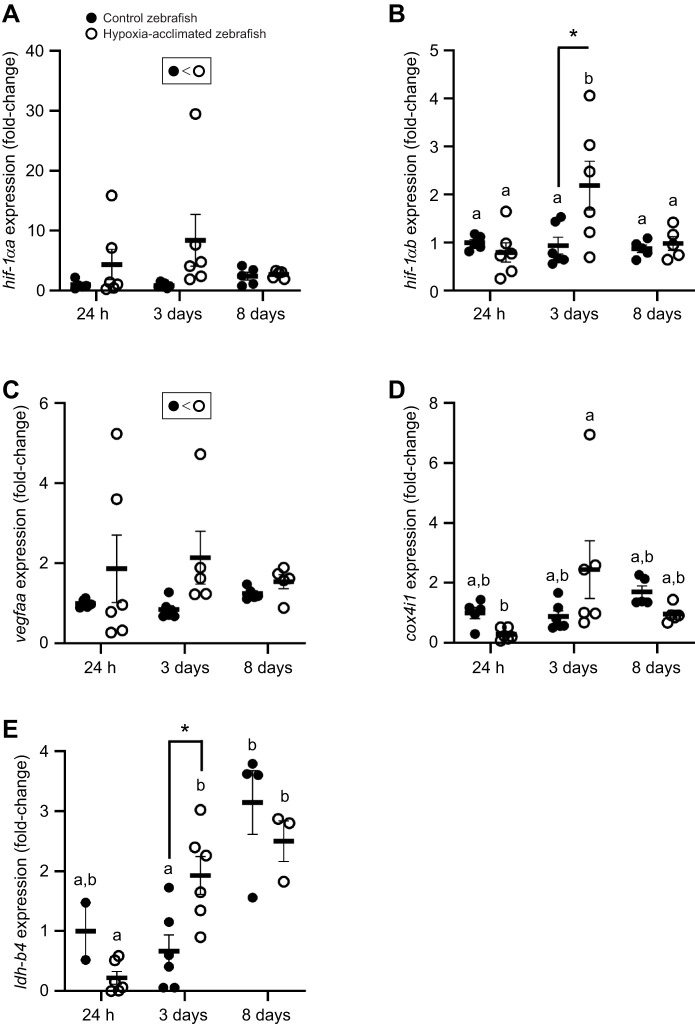
**Influence of hypoxia acclimation on the expression of marker genes for the hypoxia response, angiogenesis, mitochondrial activity and anaerobic metabolism.** (A) *hif-1αα*, (B) *hif-1αb*, (C) *vegfaa*, (D) *cox4i1* and (E) *ldh-b4*. Significant differences (*P*<0.05; two-way ANOVA) are depicted using an asterisk (between control and acclimated on sampling day), symbols (overall acclimation effect) or lowercase letters (within treatment group between sampling days). Data are plotted as means±s.e.m., and individual data points are shown.

There were no interactive effects resulting from the analysis of *hif-1αa* expression (*P*=0.107). Hypoxia acclimation was shown to have a significant effect on the expression of *hif-1αa* in the heart (two-way ANOVA, *P*=0.019). *hif-1αa* expression was shown to not be affected by time (two-way ANOVA, *P*=0.271) (Table 1, Fig 5A).

**Table 1.** Influence of hypoxia acclimation on the expression of marker genes for the hypoxia response, angiogenesis, mitochondrial activity and anaerobic metabolism

Gene	24 h		3 days		8 days	
	Control	Acclimated	Control	Acclimated	Control	Acclimated
*hif-1αα*	1±0.31^1^	2.09±1.2^2^	0.94±0.19^1^	9.70±4.58^2^	2.42±0.65^1^	2.72±0.27^2^
*hif-1αb*	1±0.07^a^	0.63±0.13^a^	0.84±0.16^a,^*	2.02±0.54^b,^*	0.88±0.08^a^	0.99±0.14^a^
*vegfaa*	1±0.47^1^	1.19±0.62^2^	0.87±1.10^1^	2.14±0.60^2^	1.24±0.06^1^	1.54±0.17^2^
*cox4i1*	1±0.19^a,b^	0.30±0.09^b^	0.90±0.21^a,b^	2.80±1.00^a^	1.70±0.21^a,b^	0.96±0.13^a,b^
*ldh-b4*	1±0.30^a,b^	0.27±0.11^a^	0.79±0.27^a,^*	2.14±0.26^b,^*	3.14±0.47^b^	2.50±0.27^b^

Data are shown as means±s.e.m. All values are fold-change in expression relative to the control value measured at 24 h. Significant differences (*P*<0.05; two-way ANOVA) are depicted using numbers (overall acclimation effect, with values that are indicated with a 1 being lower than those indicated with a 2); an asterisk (between control and hypoxia acclimated on a sampling day); or lower-case letters (within treatment group between sampling days). *N*=5–6. For raw data, see Dataset 1.

#### hif-1αb

There was a significant interactive effect resulting from the analysis of *hif-1αb* expression (two-way ANOVA, *P*=0.024). Hypoxia acclimation did not have a significant effect on the expression of *hif-1αb* in the heart (two-way ANOVA, *P*=0.088). However, *hif-1αb* expression was shown to be significantly affected by time (two-way ANOVA, *P*=0.027). At the 3 day time point, expression of *hif-1αb* in the hearts of hypoxia-acclimated zebrafish was higher than that in control fish (*P*=0.020) (Table 1, Fig 5B).

#### vegfaa

There was not a significant interactive effect resulting from the analysis of *vegfaa* expression (two-way ANOVA, *P*=0.605). Hypoxia acclimation was shown to significantly affect the expression of *vegfaa* in the heart (two-way ANOVA, *P*=0.047). Compared with those of the control fish, the hearts of hypoxia-acclimated fish had higher levels of *vegfaa* expression by 2- to 4-fold on average, with pronounced elevation at the 24 h and 3 day time points. *vegfaa* expression was consistent through time (two-way ANOVA, *P*=0.969) (Table 1, Fig 5C).

#### cox4i1

There was a significant interactive effect resulting from the analysis of *cox4i1* expression (two-way ANOVA, *P*=0.023). Exposure to chronic hypoxia was not shown to significantly affect the expression of *cox4i1* in the heart (two-way ANOVA, *P*=0.920). There were no significant differences in *cox4i1* expression between treatment groups at any time point (*P*>0.05) (Table 1, Fig 5D).

#### ldh-b4

*ldh-b4* expression in the heart was shown to be affected by time (two-way ANOVA, *P*<0.0001) and there was a significant interactive effect in the model (two-way ANOVA, *P*=0.0071). However, acclimation to hypoxia had no effect on the expression of *ldh-b4* in the heart (two-way ANOVA, *P*=0.859). Expression of *ldh-b4* in the hearts of hypoxia-acclimated zebrafish was significantly higher than that in control fish at the 3 day time point (*P*=0.046), and there were significant increases in *ldh-b4* expression over time in hypoxia-acclimated fish from 24 h to 3 days (*P*=0.0039) and in control fish from 3 days to 8 days (*P*=0.0002) (Table 1, Fig 5E).

## DISCUSSION

The result of the LOE experiment suggests that chronic hypoxia exposure prepares zebrafish for a subsequent, more severe, exposure. The changes to the structure and function of the heart caused by hypoxia acclimation, characterized here for the first time, may be responsible, at least in part, for this response. More specifically, the higher 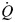 in the acclimated fish during an acute hypoxia exposure would help move blood at a faster rate through the animal and our results suggest that is due to a higher stroke volume caused by an increase in ventricular volume. Importantly, the higher end diastolic area, determined using ultrasound, supports this result. In addition to these changes to the structure and function of the heart, the measured increases in gill respiratory surface area and haematocrit, responses that have been previously reported by others, suggest a greater capacity to extract oxygen from the water and carry it to the tissues. The lack of change in cardiac collagen levels with hypoxia acclimation, measured using histological methods, suggests that there were no changes to the connective tissue content of the heart.

### Response of the cardiovascular system to hypoxia acclimation

#### Gill respiratory surface area and haematocrit

The fish gill is a highly plastic structure that responds quickly to changes in DO to help maintain efficient extraction of oxygen from the respiratory medium (Dhillon et al., 2013). The higher rate of opercular movement in the control fish, compared with that of the hypoxia-acclimated fish, at each of the measured DOs suggests that the fish are trying to move more water across the gill per unit time. Such an increase would assist in oxygen extraction as it would help maintain the difference in DO between the blood and water across the gill. Previous work has reported a similar increase in average gill ventilation rate with hypoxia exposure. For example, Vulesevic and Perry (2006) reported that acute hypoxia (∼25% air saturation) exposure increased gill ventilation rates in adult zebrafish from ∼200 to ∼350 times min^−1^. The higher rate of gill ventilation in the control group during the acute hypoxia exposure, however, does suggest that the hypoxia-acclimated fish are better able to extract oxygen from the environment. One characteristic contributing to this is the higher respiratory surface area in the hypoxia-acclimated fish. This response has been previously reported in multiple studies completed on a variety of fish species (Dhillon et al., 2013; Mitrovic et al., 2009; Sollid et al., 2003). For example, 7 days of chronic hypoxia exposure of goldfish (*Carassius auratus*) caused a decrease in ILCM (Mitrovic et al., 2009) and a similar result was reported for multiple cyprinid species after 48 h of hypoxia exposure (Dhillon et al., 2013). The increase in respiratory surface area with hypoxia acclimation complements the increase in haematocrit that was also measured in the current study. This response to hypoxia acclimation has also been reported in multiple other fish species, including rainbow trout (*O. mykiss*) (Tetens and Lykkeboe, 1981), zebrafish (Cadiz et al., 2019) and tambaqui (*Colossoma macropomum*) (Affonso et al., 2002). Interestingly, this is the first time that hypoxia acclimation has been demonstrated to affect gill ventilation rate in zebrafish. In previous studies where there was no effect of hypoxia acclimation on ventilation rate, the period of acclimation was shorter. For example, in a study with zebrafish where there was no significant change after 28 days of hypoxia acclimation (Vulesevic et al., 2006) and for a study on channel catfish (*I. punctatus*), the acclimation period was 7 days (Burleson et al., 2002). This suggests that changes to gill function increase with duration of the chronic exposure. One factor to consider regarding gill function during chronic hypoxia exposure is how an increase in cardiac output, caused by cardiac remodelling, affects the O_2_ gradient across the gill. It has been suggested that in normoxia, O_2_ movement across the gill is perfusion limited, with the relationship between oxygen uptake and the rate of blood flow through the gills being linear and positive (Daxboeck et al., 1982; Desforges et al., 2002). However, with a reduction in environmental O_2_ (hypoxia), the gradient across the gill epithelia between the water and blood decreases (Daxboeck et al., 1982). If the movement of O_2_ remains perfusion limited, an increase in blood flow rate, caused by an increase in cardiac output, would likely benefit O_2_ movement across the gill and aid in hypoxia tolerance. If, however, O_2_ movement across the gill becomes diffusion limited in hypoxia, an increase in cardiac output would not improve this. Further work is required to determine whether oxygen movement across the fish gill during hypoxia is perfusion or diffusion limited.

An increase in haematocrit is triggered during acute hypoxia exposure in response to an increase in plasma catecholamine levels (Montpetit and Perry, 1998), and work by Montpetit and Perry (1998) suggests that catecholamines are still involved in regulating this response in rainbow trout after 5 days of moderate hypoxia. The increase in haematocrit measured after 7 weeks of hypoxia exposure is also likely due to an increase in erythrocyte production, as work by Lai et al. (2006) demonstrates that chronic hypoxia exposure of trout causes an increase in erythropoietin and erythropoiesis. The higher haematocrit of the hypoxia-acclimated fish may be responsible for triggering the cardiac hypertrophy characterized in this study. This is because an increase in haematocrit causes an increase in blood viscosity which can result in an increase in the biomechanical load on the heart (León-Velarde et al., 2010). In mammalian models, increased haematocrit, such as that caused by chronic mountain sickness, leads to right ventricular hypertrophy (León-Velarde et al., 2010). This response is thought to be triggered, at least in part, by increased stimulation of mechanically gated channels that lead to the activation of the p38-JNK-ERK mitogen-activated protein kinase (MAPK) pathway (Chiquet et al., 2009; Husse et al., 2007; Okamoto et al., 2013; Reed et al., 2014; Strom et al., 2024). Activation of this pathway can lead to cardiac hypertrophy as a result of changes in the expression of specific genes and associated proteins (Shaftoe and Gillis, 2024; Sheikh et al., 2008). Importantly, work by Johnston and Gillis (2019) indicates that these pathways are activated by biomechanical stretch in trout cardiac fibroblasts. It has also been suggested that cardiac remodelling in fish, stimulated by cold acclimation, is activated by an increase in blood viscosity (Keen et al., 2016; Klaiman et al., 2011; Shaftoe and Gillis, 2024). In this instance, the increase in blood viscosity is caused by the stiffening of the lipids in the erythrocyte membranes with the decrease in temperature (Graham and Farrell, 1989; Klaiman et al., 2011).

#### Heart morphology and composition

The measurements of ventricular area, made on fixed histological sections, and end-diastolic area, measured *in vivo* using cardiac ultrasound, suggest that chronic hypoxia exposure promotes cardiac hypertrophy. These measurements are complementary as the histological measures were made on ventricles that had been maximally contracted while the *in vivo* measurements were made on hearts maximally filled with blood between beats. These results are also supported by a study from Marques et al. (2008) demonstrating that acclimation of zebrafish to 10% air saturation for 21 days caused hyperplasia in the cardiac ventricle, smaller ventricular outflow tracts and reduced lacunae within the ventricle. An increase in ventricular area, and *v*_S_ consequently, may aid the heart in maintaining 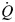 by enabling more blood to be pumped per beat. Such capacity would compensate for a decrease in heart rate during bradycardia, caused by an acute decrease in either temperature or DO.

The compact myocardium in the fish heart generates contractile force during systole. The 25% increase in compact myocardium thickness with hypoxia acclimation would therefore increase the capacity to move blood through the animal. This increased force-generating potential would help compensate for the ∼30% increase in haematocrit caused by hypoxia acclimation that, while increasing the oxygen-carrying capacity of the blood, would also, as mentioned above, increase its viscosity. Previous work has demonstrated that acclimation of steelhead trout (*O. mykiss*) to hypoxia (40% air saturation, 6 weeks) increased the contractile capacity of the contractile myocardium (Roberts et al., 2021). This supports the suggestion that hypoxia acclimation increases the functional requirements of the fish heart. Increasing the compact myocardium thickness, and therefore the capacity for force generation, may help to compensate for the effects of acute hypoxic and/or acute cold conditions on contractile function. For example, an acute decrease in temperature can impair contractile function via *Q*_10_ effects as well as a reduction in intracellular Ca^2+^, resulting in reduced contractile force. However, this remodelling response may also be species dependent, as chronic hypoxia acclimation (∼1 month) of Atlantic salmon (*Salmo salar*) produced no change in either the shape of the ventricle or the percentage of compact and spongy myocardium (Gamperl et al., 2020).

In previous work, when cardiac remodelling in zebrafish was initiated via thermal acclimation, there was a measured change in relative collagen content (Johnson et al., 2014; Shaftoe et al., 2023). Such changes in collagen content have been suggested to modulate the passive stiffness of the ventricle so as to maintain diastolic function at the new physiological temperature (Johnson et al., 2014; Johnston and Gillis, 2019; Keen et al., 2016; Shaftoe et al., 2023). The lack of change in relative collagen content in the heart with hypoxia acclimation in the current study suggests no such changes in passive biomechanical properties.

### Effects of hypoxia acclimation on *in vivo* cardiac function

When measured under control conditions (normoxia, 28°C), ƒ_H_ and 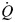 of the hypoxia-acclimated zebrafish was not statistically different from that of control fish. Importantly, however, *v*_S_ was greater in the hypoxia-acclimated fish than in the control fish under control conditions. Why this likely did not translate into an increase in 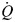 (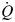
=ƒ_H_×*v*_S_) is that *f*_H_ was lower, though not statistically different, in the hypoxia-acclimated fish than in the control fish. Work from Petersen and Gamperl (2010) found that acclimation of Atlantic cod to 40% air saturation for at least 6 weeks reduced *v*_S_, causing 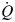 in control conditions to be significantly reduced. These differences in response may be due to the species examined. For example, zebrafish would be more routinely exposed to hypoxia in their natural environment compared with Atlantic cod, which is a cold-water species living in open water. Such species-specific differences are important to consider when predicting the consequences of hypoxia exposure due to environmental eutrophication.

The decrease in *f*_H_ of the control group with acute hypoxia exposure during the ultrasound measurements was not statistically significant, though the means differed by 30%. There was also no statistical difference between the *f*_H_ of the hypoxia-acclimated group and that of the control group when each were measured under hypoxic conditions. Here, the mean *f*_H_ of the control fish was 30% less than that of the hypoxia-acclimated group. However, the higher *v*_S_ of the hypoxia-acclimated fish translated into a higher 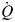
compared with that of the control fish. The 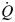 of the control fish measured during acute hypoxia exposure was also significantly less than that measured under control conditions. This decrease was likely due to a decrease in *v*_S_. Hypoxic bradycardia (a slowing of *f*_H_) caused by an increase in vagal tone leading to increased cholinergic activity is a common response in fish to an acute decrease in DO (Farrell, 2007). Evidence for this is that injection of multiple fish species with atropine, a muscarinic receptor antagonist, reduces or eliminates hypoxia-induced bradycardia (Perry and Desforges, 2006; Stecyk et al., 2020; Vornanen and Tuomennoro, 1999).

While the decrease in *f*_H_ of the control fish with acute hypoxia exposure was not statistically significant, the significant decrease in 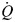 would translate into a reduction in the rate of blood movement through the animal. This bradycardic response with acute hypoxia exposure appears to be species specific and likely correlates with relative hypoxia tolerance, as hypoxia-tolerant fish, such as common carp (Glass et al., 1991), crucian carp (Stecyk et al., 2020) and air-breathing fishes (Damsgaard et al., 2020), lack this response. It has been suggested that the lack of a bradycardic response in crucian carp is because the energy requirements of normal heart function can be supported through glycolytic pathways during hypoxia exposure (Stecyk et al., 2020).

### Effects of hypoxia acclimation on whole-animal response to acute hypoxia exposure

The results from the LOE experiments indicate that the acclimated fish were more tolerant of hypoxia than control fish. The changes to the cardiovascular system, detailed above, would help ensure oxygen delivery to the tissues when environmental levels of DO become limiting. The extent, and energetic cost, of these changes to the multiple components of the cardiovascular system indicates how significant a challenge a decrease in environmental oxygen is to the maintenance of physiological activity. It is however important to put the difference in DO at LOE into context. In natural environments, such as the Central Basin of Lake Erie, DO depletion rates have ranged from 2.65 to 4.70 mg O_2_ l^−1^ month^−1^ over the last 20 years (U.S. EPA, 2023). Importantly, this area of Lake Erie goes anoxic in late summer. Using these rates, the difference in DO at LOE between the control and hypoxia-acclimated fish translates to 23–41 h of survival. While this difference may not seem significant, it is important to remember that while the LOE measurements reflect a physiologically relevant parameter measured *in vivo*, they are made under non-physiological conditions where the rate of DO depletion is accelerated and constant.

The results of the current study demonstrating that hypoxia acclimation increases the capacity of zebrafish to maintain physiological function during an acute hypoxia exposure are supported by previous studies. For example, Gilmore et al. (2019) report that acclimation of juvenile Murray cod (*Maccullochella peelii*) to hypoxia (∼50% air saturation) for 7 days increased performance in a LOE test like that used here. Similarly, Borowiec et al. (2020) found that killifish (Family Fundulidae) acclimated to hypoxia (∼10% air saturation) for 28 days also performed better in an LOE trial. Finally, Rees et al. (2001) demonstrated that acclimation of zebrafish to non-lethal hypoxia (10% DO) for 48 h increased survival time in a subsequent and more severe hypoxia exposure (5% air saturation).

### Molecular response to the first week of hypoxia exposure

Hypoxia inducible factor 1 (HIF-1), a heterodimeric DNA-binding complex, is the primary organizer of the cellular response to hypoxia exposure (Weidemann and Johnson, 2008). This protein is composed of two subunits, HIF-α and HIF-β. Under normoxic conditions, HIF-α is continuously turned over, preventing formation of the complex (Jewell et al., 2001). However, with a decrease in cellular oxygen levels, HIF-α becomes stabilized, enabling it to increase in concentration and dimerize with HIF-β (Jiang et al., 1996). This leads to the transcription of genes involved in the cellular hypoxic response (Jewell et al., 2001). In the current study, the increase in the transcript levels of *hif-1αa* at all sampling points, starting at 24 h, as well as *hif-1αb* at day 3, suggests that the HIF pathway is being upregulated quickly to enhance the cellular response to hypoxia exposure. (*hif-1αa* and *hif-1αb* are paralogues of *hif-1α* found in zebrafish; Mandic et al., 2020.) This molecular response would potentially provide more HIF-α protein to form the HIF-1 complex. Mandic et al. (2020) have suggested that one consequence of an increase in the expression of HIF-α in zebrafish with hypoxia acclimation is an increase in oxygen uptake and transport capacity, resulting in an increased performance in LOE trials. Work by others demonstrates that hypoxia exposure rapidly (within a few hours) increases *hif-1α* expression, the levels of HIF-α protein, as well as expression of its downstream target genes in the fish myocardium (Imbrogno et al., 2014; Parente et al., 2013; Rissanen et al., 2006). The *hif-1αb* transcript has been linked to increased hypoxia tolerance in cyprinid fish (carp, goldfish and zebrafish) (Elks et al., 2015). The results of the current study support this hypothesis.

One of the genes regulated by HIF-1 is *vegfaa*, a gene involved in regulating angiogenesis and muscle hyperplasia in the zebrafish heart (Karra et al., 2018; Weidemann and Johnson, 2008). The increased expression of this transcript with hypoxia acclimation suggests an increase in angiogenesis and may be associated with the measured cardiac hypertrophy in the acclimated fish. Previous studies have also demonstrated that hypoxia exposure of salmon (*S. salar*) (Yu et al., 2008) and Nile tilapia (*Oreochromis niloticus*) (Zhao et al., 2014) can lead to an increase in the expression of *vegf.* In the study with Nile tilapia, the resultant data suggested a link between the expression of *vegf* and alterations in oxygen carrying capacity, anaerobic metabolism and antioxidant enzyme activity (Zhao et al., 2014). Altogether, this suggests that *vegfaa* is an important regulator of cardiac remodelling in fish that can occur in response to hypoxia acclimation.

The transcript for COX4i (*cox4i1*) was used as a marker for aerobic capacity while *ldh-b4* was used as a marker for anaerobic metabolism. The lack of change in the levels of *cox4i1* suggests no changes to aerobic capacity over the period sampled. The higher level of *ldh-b4* expression at day 3 of hypoxia exposure suggests that there may be an increased expression of proteins involved in anaerobic pathways.

### Conclusion and perspectives

This study demonstrates for the first time that zebrafish can respond to hypoxia acclimation with ventricular hypertrophy. This response and resultant increase in *v*_S_, coupled with the increase in haematocrit, would enhance oxygen transport through the animal. The changes to the cardiovascular system with hypoxia acclimation, while increasing the capacity to transport O_2_, also have an energetic cost. This is an important consideration when working to better understand the consequences of environmental eutrophication, and climate change in general, on natural fish populations. For example, animals that display phenotypic plasticity in response to a change in environmental conditions may become increasingly challenged as said conditions become more stochastic. This idea is supported by work demonstrating that the body condition of zebrafish that had been rewarmed following cold acclimation was lower than that of control fish (Shaftoe et al., 2023). So, while remodelling of the cardiovascular system to a change in environmental conditions provides a benefit in the moment, reversal of such a response with a subsequent change also has a cost. These costs may add up if environmental conditions, including DO and/or environmental temperature, fluctuate multiple times throughout a season or year.

## Supplementary Material

10.1242/jexbio.251606_sup1 Supplementary information

Dataset 1. Raw data for Figs 1-3 and Table 1.
