# Supplementary material for: Integrated response of the zebrafish (Danio rerio) cardiovascular system to hypoxia acclimation
Source: J Exp Biol. 2025 Nov 20;228(22):jeb251606. doi: 10.1242/jeb.251606 (PMC12669836; doi:10.1242/jeb.251606)
Supplement: Supplementary information [file jexbio-228-251606-s1.pdf]

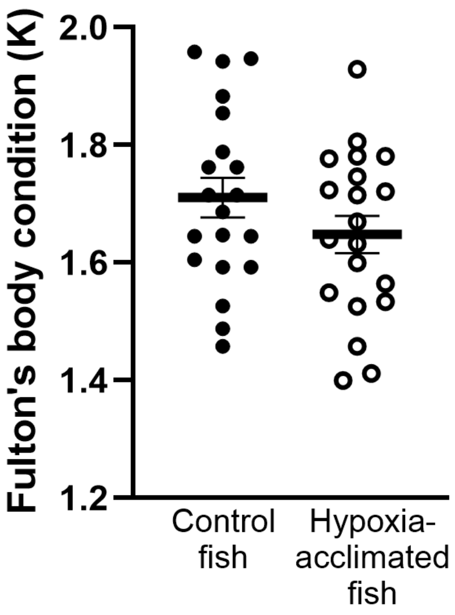

Fig. S1. Influence of hypoxia acclimation on the Fulton's body condition factor (K) of adult zebrafish (*Danio rerio*). Data are plotted as means ± S.E.M., and individual data points are shown. There is no difference between means ($p > 0.05$).

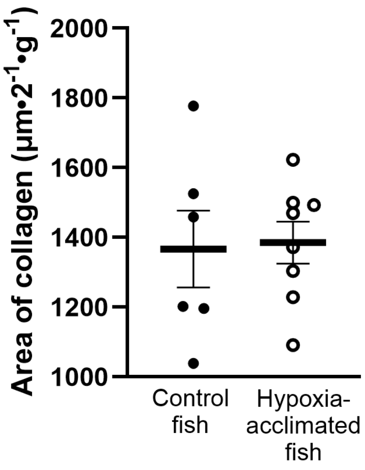

Fig. S2. Influence of hypoxia acclimation on the collagen content of the zebrafish (*Danio rerio*) heart. Area of collagen, measured histologically and normalized to body mass. Data are plotted as means ± S.E.M., and individual data points are shown. There is no differences between means ($p > 0.05$).

Journal of Experimental Biology • Supplementary information

Table S1. Information on primers used for qPCR.

Gene	Sequence (5'-3')	Primer concentration (nM)	Efficiency (%)	R^2	Genbank reference
hif-1αa	F: ACTCATCCGTGT GACCATGA R:ACCTTCCAGG AGGCAGATTT	200 nM	97%	0.99	NM_001308 559.1
hif-1αb	F: GCCTATCTCCTC TCACACCC R:CGTAATCCAT GGGGCTGTTG	300 nM	100%	0.99	NM_001310 042.1
vegf	F: CGCCGCTATCA CCTACAAAT R: GGAGGTTGTCT GGCTCTCAG	200 nM	103%	0.98	AF016244.1
cox4i1	F: CACATGGAGTT GCGAAGGTC R: CTCTTTGGACA GTGCAGCC	100 nM	99%	0.98	NM_214701. 1
ldh-b4	F: GTACTGCTTCG GGAACTTGC R:GCAGTCACCA CCACAATACG	100 nM	97%	0.99	AF067202.1
ef1α	F: GTGCTGTGCTG ATTGTTGCT R:TGTATGCGCT GACTTCCTTG	300 nM	97%	0.99	L47669.1
rpl8	F: ATAGTCTGCTGT CTGGAGGAG R: TCGGGATTGTG GGAAATAACG	100 nM	97%	0.99	NM_200713

Dataset 1. Raw data for Figs 1-3 and Table 1.

Available for download at
https://journals.biologists.com/jeb/article-lookup/doi/10.1242/jeb.251606#supplementary-data
